# The Link Between Contextual Poverty and Academic Achievement: Evidence Using Panel Data From a Lower‐Middle‐Income Country

**DOI:** 10.1111/1468-4446.13208

**Published:** 2025-03-26

**Authors:** Mobarak Hossain

**Affiliations:** ^1^ London School of Economics and Political Science (LSE) London UK

**Keywords:** Bangladesh, educational inequalities, locality, subdistrict, urban‐rural

## Abstract

The association between contextual poverty and educational achievement is not well‐researched in lower‐income countries. This paper investigates this link and examines how it varies between urban and rural school locations in Bangladesh, acknowledging the dual urban‐rural dynamics of the country. Analyses based on original school‐level longitudinal data, encompassing over 90 per cent of secondary schools in Bangladesh, demonstrate that subdistrict‐level educational poverty (measured as the proportion of adults with education below the primary level) has a stronger and significantly negative association with achievement at the secondary level compared to economic poverty (measured as the percentage of people under the national poverty line). This negative association is starker for the ‘science’ academic stream, which necessitates supplementary private tutoring. I argue that in poorer local areas, pupils are less likely to encounter successful role models in science fields, experience a shortage of qualified instructors, and face difficulties in securing additional resources for science subjects due to poverty. Furthermore, urban areas generally exhibit higher achievement levels, reflecting a greater proportion of educated individuals and role models. However, urban achievement experiences a sharper decline with increasing educational poverty, likely due to structural inequalities such as informal settlements and unequal access to quality schools. In contrast, rural areas show less sensitivity to educational poverty, possibly due to the ‘scarcity effect’ of role models, where the limited presence of role models exerts a disproportionately positive influence on aspirations, even in high‐poverty contexts.

## 1 Introduction

What factors drive educational disadvantages at the individual and school levels in low‐ and middle‐income countries (LMICs)? To answer this question, existing research has primarily investigated the role of family socioeconomic status (SES) and school resources (Bouhlila 2015; Silva et al. 2021; Torche 2019). However, little is known about the influence of poverty in local areas or contexts on educational achievement and the mechanisms underlying this relationship in LMICs. Children's socialisation processes in deprived localities and communities may adversely affect their learning. Past research from advanced economies provides conclusive evidence that poverty in neighbourhoods or smaller geographical units has significantly negative associations with various educational outcomes (e.g., Chetty et al. 2016; Crane 1991; Harding 2003; Nieuwenhuis and Hooimeijer 2016; Wodtke et al. 2011).

Addressing the lack of empirical evidence from LMICs, this study examines two main questions in the context of Bangladesh, using school‐level analysis. First, to what extent is poverty at the local or contextual level (subdistricts) associated with learning achievement in schools? Here, achievement refers to schools' performance in national exit examinations. Second, to explore potential mechanisms, I investigate whether the association between local poverty and achievement varies by school location (urban or rural), as rurality remains a predominant feature of Bangladesh, as in many other LMICs. The significant presence of the rural population in LMICs makes it vital to examine how poverty's effects in rural areas differ from those in urban areas, a topic often overlooked in the current literature.

To address these questions, I draw on data from Bangladesh, categorised as a lower‐middle‐income country (World Bank, n.d.‐c) with one of the world's largest education systems. I use a unique, untapped longitudinal dataset covering more than 90% of secondary schools across all districts and nearly all subdistricts from 2011 to 2019. This dataset is linked to subdistrict‐level poverty data, with local areas defined as subdistricts—the lowest administrative unit at level 3 responsible for implementing public policies (following divisions at level 1 and districts at level 2). The novelty of this study lies in its use of a rich longitudinal dataset, addressing a common barrier to researching this topic in LMICs.

Despite a wealth of literature linking contextual characteristics to students' learning achievement, research from LMICs remains scarce. Discussions on educational inequalities in LMICs have largely focussed on the extent to which family SES and school characteristics explain children's educational outcomes. Although past research is inconclusive, it suggests that school resources and characteristics play a dominant role in LMICs (Heyneman and Loxley 1983). Conversely, some recent studies highlight the importance of family SES (Baker et al. 2002; Crouch et al. 2021; Jones and Schipper 2015; Silva et al. 2021).

However, limited attention has been given to neighbourhood or local area characteristics in LMICs, where schools are embedded, and children grow up socialising. Learning achievement may be significantly influenced by the surrounding environment rather than just SES or school characteristics. Additionally, the level of absolute poverty in LMICs is generally higher than in high‐income countries (HICs) (Alkire et al. 2015). Hence, the dynamics linking local poverty and learning achievement may differ substantially between these contexts. For example, one difference may stem from urban‐rural disparities, as rurality is a predominant feature in many LMICs. In Bangladesh, 62% of the population lived in rural areas in 2020, compared to 17% in the United States and 19% in HICs (World Bank, n.d.‐b).

Moreover, empirical findings on neighbourhood poverty and educational outcomes mostly derive from Western, highly urbanised societies, particularly the US. Mainstream sociological literature examines neighbourhood poverty and its effects from an urban perspective (Ainsworth 2002; Duncan 1994; Garner and Raudenbush 1991; Wilson 2011, 2012). Recent work from the UK, another highly urbanised country, on local context and intergenerational social and educational mobility has not examined urban‐rural differences (Breen and In 2024a, 2024b). However, many developing societies in LMICs are less urbanised and some are predominantly agrarian. While relevant, theories derived from HICs may not fully apply to LMICs, particularly in rural contexts. Drawing a cross‐sectional analysis from 30 LMICs, Huisman and Smits (2009) find that rurality, a district‐level contextual factor, is negatively associated with children's school enrolment. Specifically in Bangladesh, most secondary schools are located in rural areas (EMIS, n.d.). Although rural areas are generally more disadvantaged because of limited access to employment opportunities and other economic activities, urban towns and cities show high and growing levels of inequality, evidenced by the rise of informal settlements and slums (United Nations 2020). Approximately 40% of the urban population resides in slums with informal settlements and substandard living conditions (Hasan et al. 2024), where literacy rates are significantly lower compared to other areas (BBS 2024). With overall lower levels of urban poverty compared to rural areas but greater inequality within urban areas, this study provides a novel empirical contribution by examining the context of Bangladesh.

Research in this area has also overlooked the differences in academic achievement across streams. There are three academic streams—arts/general, business, and science—that begin at the secondary level in Bangladesh. As I explain in the Theoretical Framework section, contextual poverty may influence students in different academic streams differently. With these societal differences in mind, I summarise the research questions below.

1. How far is poverty in local (subdistrict) areas associated with decreasing learning achievement in Bangladesh? A relevant sub‐question is: does this association, if any, vary by academic subject streams: arts/general, business, and science?
2. To what extent does the link between local (subdistrict‐level) poverty and achievement differ between urban and rural areas?

## 2 Theoretical Framework

### 2.1 Defining Localities

Neighbourhoods are clusters of residences with some common spatial attributes such as building type and design, roads and sidewalks, demographic and class characteristics, and networks (Galster 2001). Since the size of subdistrict territories (known as *Upazilas* and urban *thanas*) in Bangladesh is considerably large, consisting of over 500 in total in the country (Hossain and Hossen 2020), these areas cannot be strictly seen as neighbourhoods. Hence, by ‘localities’ or ‘local areas’, I refer to level‐3 administrative units or ‘macro‐neighbourhoods’. I use localities, local areas, local contexts, and subdistricts interchangeably in the paper. The boundaries of local contexts have not been explicitly discussed in the literature and broader administrative boundaries have been used in the recent literature (Breen and In 2024a).

Subdistricts are the focal points of implementing public policies at the grassroots level in Bangladesh (Osman et al. 2015). As most secondary schools in Bangladesh are partially (especially teachers' salaries) or fully funded by the government (Al‐Samarrai 2007), these local units may partly influence how education policies are implemented across subdistrict areas. This makes the variation in educational outcomes between these macro localities important to look at. The lowest administrative tier is level 4 or ‘unions’ in the country, which do not have responsibilities related to education provision.

Despite having aggregated subdistrict‐level data, I frame the theoretical understanding of the link between local poverty and achievement using neighbourhood literature. This is for two reasons. *First*, according to the Education Management and Information System (EMIS), nearly 85% of secondary schools in Bangladesh are in rural areas (EMIS, n.d.). While rural inhabitants are not homogeneous, rural neighbourhoods in Bangladesh, like many other LMICs, tend to share some common characteristics such as low education level, agriculture‐dominated employment, and less developed physical infrastructures such as roads and transports (Khan 2001; Meemken and Bellemare 2020). This means the literature about neighbourhood poverty can be suitable to theorise the role of local areas or aggregated administrative units in education. The spatial homogeneity may somewhat be similar in less urbanised towns as well. However, I recognise that the presence of more heterogeneous spatial units in some large cities, especially in Dhaka, the capital, and Chittagong, the second largest city, makes it less suitable to theorise aggregated units by neighbourhood literature. Due to the data limitation, I could not further disaggregate spatial units, which is the *second* reason for using neighbourhood literature for analysing the role of macro‐administrative units.

### 2.2 Linking Local Poverty to Achievement: Theoretical Models

In this section, I discuss theories from the sociological and social psychological literature on *collective socialisation* and *social capital* to explain the mechanisms through which poverty in local areas can be linked to learning achievement in LMICs.

*Collective Socialisation.* The ‘role model effect’ has been used in sociological literature to show how macro contextual factors influence collective socialisation processes as children learn about their role models by socialising with others outside the home. Communities with higher concentrations of educated adults provide more visible role models for younger generations to be more aspirational about their education and careers. They also work hard in schools, which they adopt from adult models. By contrast, disadvantaged neighbourhoods with high unemployment and low levels of education may not be conducive to children's education (Harper et al. 2003; Wilson 2011). Kemper (1968) argues that certain reference groups may be more conducive to fostering individual achievements including in education. Ainsworth (2002) finds collective socialisation has a strong mediating effect on learning achievement. The role model effect theory is also supported by the social learning theory by Bandura and Walters (1963), which suggests that individuals in a community learn through observation, imitation, and modelling. Learning as a cognitive process can be influenced by social contexts. Hence, poverty in a community may limit the visibility of positive learning behaviours, reducing motivation and achievement in subsequent generations. For instance, in many LMICs, poor local areas may have lower employment opportunities, which may negatively affect the perceived benefits of receiving an education (Huisman and Smits 2009).

I expect that this mechanism may also apply to the context of LMICs including Bangladesh since many of these societies have collectivist cultures (Huang 2006). Since people have certain bonds in the same neighbourhood or local area, children are more likely to follow their adults as role models. Consequently, advantaged neighbourhoods with more highly educated and higher‐SES inhabitants would offer better role models, which could encourage young people to perform better in school (Huang 2006).

*Social Capital*. Social capital may provide children with networks of relationships that help them access resources and opportunities (Bourdieu 1986). In disadvantaged communities, poverty may be associated with weaker networks of educated individuals and high‐status professionals who can provide them guidance, mentoring, and help access resources, creating a cycle of disadvantage. In contrast, in advantaged neighbourhoods, children are more likely to have access to helpful networks of adults who can connect them with opportunities and resources beneficial to career advancement (Wilson 2011). This process is also known as social capital outside the family (Coleman 1988), which is built through parents' relationships with community institutions by building ‘norms, interpersonal trust, social networks, and social organisation’ (Coleman 1988, *p*. S96).

I argue that neighbourhood effects, through social capital and collective socialisation, may apply to LMICs and, specifically, to Bangladesh. In predominantly collectivist societies, social ties between individuals and groups are often stronger (Liu et al. 2019; Yamagishi et al. 1998). These social networks may be further reinforced by a sense of fraternity derived from religiosity (Gurrentz 2014), particularly in a highly religious country like Bangladesh, where fewer than 0.2% of the population identifies as non‐religious (Lynch et al. 2022). Hence, privileged neighbourhoods can offer children better access to resourceful networks, which can facilitate opportunities such as internships, graduate job placements, or knowledge acquisition through study tours.

The influence of collective socialisation processes and social capital on educational achievement would depend on how advantaged or disadvantaged neighbourhoods or local areas are. I rely on two indicators to capture the disadvantages in local areas: (i) educational (share of adults with certain levels of education) and (ii) economic poverty (share of population under the national poverty line). Socialisation processes in educated networks would provide children with informational capital encouraging them to continue their education and improve their achievement levels. Contrarily, local areas with fewer educated adults may have detrimental effects on younger individuals' education. Similarly, a lower poverty level in a neighbourhood means children are less introduced to poverty‐related social problems such as unemployment and disparity (Amara and Jemmali 2018). If children are not used to seeing success stories and examples from their older peers, they might not feel encouraged to demonstrate academic effort (Ainsworth 2002; Harper et al. 2003; Massey et al. 1991; Wilson 2012).

### 2.3 Differences by Streams

Local poverty may likely influence pupils' learning achievement based on their specialisation. Students can freely choose from three different streams—arts, business, and science—from grade 9 or ‘middle‐secondary’ to 12 or upper‐secondary.^1^ Science subjects typically involve physics, chemistry, biology and advanced mathematics while business includes subjects such as accounting and commerce, and arts/general stream emphasises social sciences, literature, and history. Students focussing on science subjects tend to take more private supplement tutoring, which requires a substantial amount of monthly educational spending from a household (Mahmud and Bray 2017) similar to other countries in Asia (Bray 2006; Bray and Kwok 2003). More crucially, better results in science subjects also depend on the availability of qualified teachers in the community, as well as inspiration and guidance from the older cohort. Hence, following the ‘role model effect’ theory in collective socialisation, greater educational poverty may affect science achievement more negatively compared to arts and business streams, as poverty and the unavailability of qualified teachers would create barriers to taking supplementary tutoring.

Besides, almost all secondary schools in Bangladesh are classified as private, often categorised as semi‐government, as the government partly funds teachers' salaries in the majority of those schools (BANBEIS, 2023; UNESCO, 2021). However, these schools retain autonomy in setting fees, which widely vary (JICA, 2023). Most secondary schools in the country are low‐fee private schools, making them widely accessible. Additionally, stipends are provided for female students, particularly in rural areas where the majority of these schools are located (UNESCO 2021). Students are free to choose their school and stream. Upon completing the middle‐secondary level (Grade 10), students apply for admission to the upper‐secondary level (Grades 11 and 12), typically based on their exit exam results rather than an admissions test. All schools follow a uniform core curriculum.

### 2.4 Local Poverty and Achievement: Urban‐Rural Differences

In Bangladesh, the rural population accounted for 62% of the total in 2020 (World Bank, n. d.‐b), while hosting 85% of all secondary schools (EMIS, n.d.). The spatial and socioeconomic features of urban and rural areas in Bangladesh are distinct, shaped by differing economic structures and social environments. Rural areas primarily depend on agriculture (Borras Jr 2009), consisting of landowners, tenants, and landless unskilled workers (Khan 2001). While heterogeneous, the spatial distribution of these groups across rural areas is likely more homogenous compared to the diverse socioeconomic structures of urban areas. In urban regions, labour markets are more varied, housing inequalities are pronounced, and disparities in wealth and living conditions are striking (Zhang 2016). For instance, slums and shanty housing often coexist alongside high‐rise buildings and affluent neighbourhoods, illustrating the high degree of spatial and socioeconomic inequality.

These urban‐rural differences can be conceptualised through collective socialisation and social capital theories. In rural areas, the relative homogeneity of socioeconomic groups fosters stronger bonding social capital, characterised by close‐knit networks, shared norms, and mutual support within communities (Coleman 1988). Resource‐poor rural communities may rely on collective socialisation processes that reinforce shared aspirations and educational values. For example, the scarcity of role models in rural areas may enhance their influence, as individuals with educational attainment stand out as visible examples to emulate. This aligns with theories suggesting that scarce, yet impactful role models can stir higher aspirations in otherwise disadvantaged areas. Hence, we may see rural achievement is less sensitive to increased poverty rates compared to urban achievement.

Urban areas in Bangladesh are marked by greater heterogeneity, both economically and socially. For instance, 40% of the urban population lives in slums with significantly lower literacy rates (BBS 2024; Hasan et al. 2024). This diversity may lead to weaker bonding social capital and fragmentation of collective socialisation processes. Urban localities, particularly in disadvantaged neighbourhoods such as slums, may lack role models, cohesive social norms, and trust networks that support educational attainment. Instead, urban schools in disadvantaged areas face compounded challenges due to high concentrations of low‐skilled workers, inadequate housing, and insufficient access to quality schools. Hence, the achievement may be more sensitive to increased poverty. This aligns with Bourdieu's theory of social capital, which emphasises that unequal access to resources through social networks perpetuates educational inequalities (Bourdieu 1986). In urban settings, the absence of cohesive networks and visible role models may exacerbate the influence of neighbourhood poverty on educational outcomes.

Given these notable differences, I hypothesise that the relationship between local poverty and educational achievement will vary by urbanicity. While urban schools are expected to perform better on average than rural schools, the association between neighbourhood disadvantages and achievement will be stronger in urban schools.

## 3 DATA and Methods

### 3.1 Data

I compiled original and comprehensive school‐level panel data for a decade from 2011 until 2019 from Education Management and Information System (EMIS) Bangladesh by web scrapping raw and unorganised data (EMIS, n.d.). This administrative data required substantial time for cleaning and organisation. The final dataset combines indicators downloaded in different files and then merged using a unique institution (school) id. The data contains more than 90% of schools from all 64 districts in Bangladesh. The primary unit of analysis is school and year.^2^ I keep schools that have data for at least two rounds out of 9 years.

As discussed, I refer to localities by subdistricts (*Upazilas* and metro *thanas*). There are over 500 subdistrict units in the country combining 492 Upazilas and some metro thanas (Hossain and Hossen 2020). After cleaning and sorting data based on the above criteria, the study covers 505 of these subdistricts or localities at the lower‐ and middle‐secondary levels and 499 at the upper‐secondary level. Neighbourhood data are typically available from small local units in a few high‐income countries. However, in the context of Bangladesh, there is no such data available. Nevertheless, this would still enable me to capture the variation across subdistricts or the focal points of public policy implementation at the local level.

Since data came in raw format and were not used before, I conducted various robustness checks to test the quality of the data. For instance, I manually matched the exit exam results for some well‐known schools in the dataset with the information available on school websites. This confirmed the high quality of the data. After cleaning and constructing variables, the final dataset includes 23,856 schools that have lower‐secondary grades, 24,275 schools with middle‐secondary grades and 5693 schools that have upper‐secondary schools. These represent more than 90% of schools in Bangladesh.^3^ The reason the number of lower and middle‐secondary schools is higher is that many schools in the country have these levels combined in the same institution and, sometimes, together with the primary level. However, upper‐secondary schools are predominantly separate with a larger pool of students and sometimes combined with some tertiary educational institutions.

### 3.2 Variables

#### 3.2.1 School Level

***Dependent variable: Educational achievement.*** This variable indicates the proportion of high‐performing students in a school. This is measured at the school level by the percentage of students who achieved 80% marks or an A+ grade per school‐year in three different secondary‐level exit exams. These are (1) Junior School Certificate (JSC) or equivalent^4^ at the end of grade 8 or lower‐secondary, (2) Secondary School Certificate (SSC) or equivalent^5^ at the end of grade 10 or secondary (middle), and (3) Higher Secondary Certificate (HSC) or equivalent^6^ at the end of grade 12 or upper‐secondary level. Hence, I use three outcome variables in the study covering the whole secondary education system in Bangladesh. However, since the results are consistent across all these outcomes, I mainly present findings from the middle‐secondary level, with the remaining results being provided in the online supplement. I use the highest grade level as an achievement indicator since the motivation is to investigate which schools perform the best in localities and if aggregated poverty level has an influence on this. However, I run robustness checks with other cut‐off points such as the percentage of students scoring 70%–79% instead of 80% in the exit exams (see the robustness section). Finally, the sample does not include the vocational track where 8.6% of students participated in 2019 at the upper‐secondary level (UIS, n.d.). The following are the school‐level independent variables. Descriptive statistics are presented for this and all other variables in Table 1.

**TABLE 1:** Descriptive statistics.

	Lower‐secondary		Middle‐secondary		Upper‐secondary	
	Mean	SD	Mean	SD	Mean	SD
% Of students achieving A+ or 80%–100% marks in a school year in exit exams	4.47	8.94	4.90	13.19	2.90	8.87
% Of children received stipends	30.48	10.07	29.21	10.88	28.46	11.49
Educational poverty (% with less than primary education) (2011)	51.69	10.49	51.07	10.75	50.28	11.60
Economic poverty (% living under the national poverty line) (2011)	32.67	13.76	32.43	13.94	31.75	14.12
Economic poverty (% living under the national poverty line) (2016)	28.52	15.17	27.55	15.10	27.41	15.44
Ratio of households without toilets (ref: no toilets) (2011)	8.76	9.40	8.14	8.98	8.38	9.24
Total sub‐district population (log)	12.56	0.49	12.57	0.51	12.58	0.53
Ratio of schools with a computer? (ref: no computers)	29.40		29.42		29.72	
Ratio of urban schools (ref: rural)	12.45		14.72		26.97	
School distance from sub‐district town (km)	9.48	6.20	9.17	6.22	7.94	6.39
School ownership and management						
autonomous	0.51		0.62		0.73	
govt	0.04		0.04		0.28	
local govt	0.22		0.28		0.37	
other	0.47		0.49		0.88	
semi govt	98.76		98.57		97.74	
School location						
Flat land	88.56		88.92		91.24	
Water‐inundated, riverbank and coastal areas	9.7		9.22		7.3	
Hilly areas (less developed communication infrastructures)	1.73		1.86		1.46	
Stream						
Arts/general	n.a		46.7		51.29	
Business	n.a		19.26		23.49	
Science	n.a		34.03		25.22	
School type by gender segregation						
Boys	0.95		1.18		0.67	
Girls	14.17		12.97		13.9	
Co‐education (combined)	83.05		83.37		83.13	
Co‐education (separate)	1.83		2.48		2.3	
General versus religious school (collapsed as a binary measure to describe easily)						
General school	63.91		79.47		30.50	
Islamic school (madrasah)	36.09		20.53		69.50	
MPO school? Yes (ref: no)	92.42		94.99		90.63	
*N* (Level 1—School year)	94,856		243,748		44,948	
*N* (Level 2—School)	23,856		24,275		5693	
*N* (Level 3—Sub‐district/Upazila)	505		505		499	
*N* (Level 4—District)	64		64		64	

*Note:* (a) MPO or schools are registered to receive ‘monthly pay order’ from the government. (b) n.a. or not applicable, as academic streams only start from the middle‐secondary level.

***Academic streams.*** JSC or the lower‐secondary level does not have any within‐school streams but SSC and HSC or the secondary and upper‐secondary levels do. The general academic stream in the latter two levels places students into (1) arts or general (e.g., history, social studies, and economics), (2) business studies (e.g., accounting, finance and banking), and (3) science (e.g., physics, chemistry, biology and advanced mathematics). All academic streams including business and science involve subjects such as language, literature and religion. Educational achievement data at the school level are separate for these three academic streams at the middle‐ and upper‐secondary levels. Hence, I control for these streams in the analysis. I will also use these streams to examine whether local poverty is associated with decreasing achievement for particular streams as explained.

***School type by gender segregation*.** This is a categorical variable indicating whether a school is for (1) boys only, (2) girls only, (3) co‐education combining girls and boys in the classroom, and (4) co‐education providing gender‐separate education.

***General versus religious school.*** This variable indicates whether the educational achievement data come from general or Islamic schools (madrasah). Each of the three secondary‐level exams has two categories indicating their exam type.

***School computer.*** This is a binary indicator signifying whether a school has computers or not. This is a time‐invariant measure as it is only available in 2019. This would still capture the financial disadvantages of schools since poorer schools may likely consistently lack this type of resource. However, while owning a computer may show schools' access to certain resources, the data do not specify whether students can use the computer.

***Schools' urbanicity.*** This is a binary indicator of whether a school is urban or rural. I use the definition of the government of Bangladesh to identify urbanicity. Using the definition by Chatterji and Yang (2016), urban areas are identified as ‘any developed areas around an identified central place with amenities such as paved roads, electricity, gas, water supply, sewerage, and sanitation; that is densely populated; where most of the population is employed in non‐agricultural sectors; and where a sense of community is well developed’ (p. 242).

In addition to this, to capture the degree of urbanicity, I also use an additional control since the distance of schools from the centre of subdistrict towns in terms of kilometres. The two indicators are different as the first one captures the urbanicity of schools regardless of their distance which is measured by the second one. Distance to the town centre may make a difference in accessing urban facilities. Moreover, the distance from the town centre does not necessarily indicate that the town is urbanised. Town centres can still be rural but have better facilities. The correlation matrix in Supporting Information S1: Table S1 suggests that the correlation between the two variables is very weak.

***School land location.*** Schools may face disadvantages based on where they are located. I further classify schools based on three such categories: whether a school is located in (a) flatlands, (b) coastal or inundated areas, and (c) hilly areas. Students and schools tend to be more disadvantageous in categories ‘b’ and ‘c’, which consist of around 11% of schools.

***School ownership.*** School ownership is a categorical variable consisting of six categories indicating the entity responsible for a school's funding and management. These are (a) private or autonomous, (b) government, (c) local government, (d) semi‐government,^7^ and (e) other.

***School type by levels included.*** School type includes four categories, whether an educational institution is (a) school (containing below 10th grade, excluding upper‐secondary), (b) college (upper‐secondary level while may also include tertiary levels),^8^ (c) school and college (all secondary levels), and (d) Islamic school or madrasah.

***School registration.*** I also control for whether a school is registered by the government in the monthly pay order (MPO) scheme or not. Hence, this is a binary variable. A school can be non‐government, but its teachers may be salaried by the government.

#### 3.2.2 Subdistrict/Local Level

***Key independent variables: local poverty.*** Aligning with the theoretical assumptions of multidimensionality of poverty, I use two measures of poverty: (1) educational and (2) economic poverty at the subdistrict or local level.

1. **Educational poverty.** The measure for poverty at the local level by education is the *proportion of adults (18 and above) with less than primary education in a locality.* The data for this variable come from Bangladesh interactive poverty maps prepared by the World Bank (2016) using the 2011 census of population and housing. This means the variable is time‐invariant as the data are only available from one time point. Past research suggests that poverty at the ecological level stubbornly persists over time, evidently for a decade (Sampson 2009; Solari 2012). Moreover, I find that the relationship between educational poverty and the economic poverty measures from 2011 to 2016 are quite similar (see Supporting Information S1: Figure S1). This means local poverty consistently predicts, although moderately, educational poverty over time.
2. **Economic poverty.** For economic poverty, I use the proportion of people living below the upper line of the national poverty line in a locality or subdistrict. Poverty is defined as the population living below the national poverty line in absolute terms. The data for this variable are also cross‐sectional. However, the data are available from two time points, 2011 and 2016. The data for 2011 come from Bangladesh poverty maps 2010 available on the World Bank's website (World Bank 2016) using the 2010 Household Income and Expenditure Survey (HIES) and the 2011 Population Census (World Bank; World Food Programme; Bangladesh Bureau of Statistics 2010). Likewise, data for 2016 come from Bangladesh poverty maps 2016. I manually extract the local level (subdistrict) poverty measures from the appendix of the poverty maps report (Hossain and Hossen 2020). Since poverty estimates for 2011 come from two sources while for 2016 from one source, the estimates are not perfectly comparable. I use both measures in separate analyses to check if I get consistent results and whether there are considerable differences because of a 5‐year time gap between the two estimates. The plotting of both measures in Supporting Information S1: Figure S2 shows a positive correlation.

National poverty lines are estimated by the status of household per capita consumption. A household is poor if its consumption is below the poverty line for the survey strata. Considering spatial differences in the expenses of purchasing basic needs, the poverty line varies across strata. This means poverty lines are adjusted in different survey rounds considering food price inflation (World Bank 2020).

I also run robustness checks using the measure of ‘extreme poverty’, which means the percentage of the population that lives below the national lower poverty line. The measure comes from the World Bank (2016) as well.

***Ratio of the households without toilets.*** Growing up without toilets and proper sanitation facilities is negatively associated with learning achievement in LMIC contexts (Spears 2012). This can affect girls' education more, in particular, when they do not have separate toilets (Glick et al. 2011). I capture this disadvantage by using the ratio of households without toilets in a locality. Supporting Information S1: Table S1 shows that this variable is not highly correlated with economic poverty.

***Total subdistrict population (log).*** The log of the total population at the subdistrict level is used as a control from the last census in Bangladesh in 2011 available from the World Bank (2016). The size of the population may affect the total ratio of achievement.

### 3.3 Statistical Models

I use four‐level multilevel models to answer the two research questions, which allow random intercepts to vary (Raudenbush and Bryk 2002). This is a suitable method for this study since the motivation is not only to look at the association between local poverty and educational achievement but also how much between‐locality (subdistrict) variance in achievement these factors can explain. In Equation 1, I examine the extent to which locality‐based poverty is associated with learning achievement in schools, the first research question. Here,

(1) $${\text{A}}_{\text{tijk}}=\alpha +\beta_{1}{\text{N}}_{\text{jk}}+\beta_{2}{\text{S}}_{\text{ijk}}+{\text{Y}}_{\text{t}}+\omega_{k}+{\text{u}}_{\text{jk}}+{\text{r}}_{\text{ijk}}+\varepsilon_{tijk}$$

*A* is the educational achievement in school *i*, at time *t*, subdistrict *j*, and district *k*. *α* is an intercept and ***β***_1_ is a vector of coefficient for time‐invariant local poverty variables ***N***_*jk*_. ***β***_2_ is a coefficient vector for school characteristics ***S***_*ijk*_ and *Y*_*t*_ is the year fixed effects. In this four‐level model, *ω*_*k*_ is the level‐4 (district) variance component (ω_*k*_ ∼ *N* (0, $\sigma_{\omega }^{2}$)), *u*_*jk*_ is the random intercept for level 3 (subdistrict or local) (*u*_*jk*_ ∼ *N* (0, $\sigma_{u}^{2}$)), *r*_*ijk*_ is the level‐2 (school) random intercept (*r*_*ijk*_ ∼ *N* (0, ${\;\sigma }_{r}^{2}$)), and ε_*tijk*_ is a level‐1 (school‐year) error term (ε_*tijk*_ ∼ *N* (0, $\sigma_{\varepsilon }^{2}$)).

I add variables step by step in the regression models. After setting out the baseline model with basic characteristics of schools as controls, I add educational and economic poverty in separate models. I also add both poverty measures in the same model and the correlation between these two poverty measures is not high (*r =* 0.29 for the 2011 poverty measures). Next, I examine whether the urbanicity of schools partly explains the relationship of poverty measures with achievement as I suggested in the theoretical framework.

Furthermore, one may argue that some of the school characteristics I use in the study as controls such as schools having computers may be directly associated with local poverty. I contend that since most secondary schools are fully or partially funded by the government (Al‐Samarrai 2007), resource distribution may not likely be notably different across local areas. To test this assumption, I run models with and without controls (including having computers in school, the school's urbanicity and distance from town, and regional disadvantages of being in water‐inundated and disaster‐prone coastal areas or hilly areas).

To investigate the second research question about whether the association between poverty and achievement differs by the urbanicity of schools, I fit Equation (2). Here,

(2) $${\text{A}}_{\text{tijk}}=\alpha +\beta_{1}{\text{N}}_{\text{jk}}+\beta_{2}{\text{U}}_{\text{jk}}+\beta_{3}\left({\text{N}}_{\text{jk}}\times {\text{U}}_{\text{jk}}\right)+\beta_{4}{\text{S}}_{\text{ijk}}+{\text{Y}}_{\text{t}}+\omega_{k}+{\text{u}}_{\text{jk}}+{\text{r}}_{\text{ijk}}+\varepsilon_{\text{tijk}}$$

in addition to Equation 1, I add the interaction of the local poverty measures ***N***_*jk*_ and urban/rural dummy *U*_*jk*_ in which ***β***_3_ is a corresponding coefficient vector. The upward or downward urban slope from the interaction effect would suggest if urban schools achieved higher or lower compared to their rural counterparts with an increasing level of poverty in the *x*‐axis.

## 4 Findings

**FIGURE 1:**
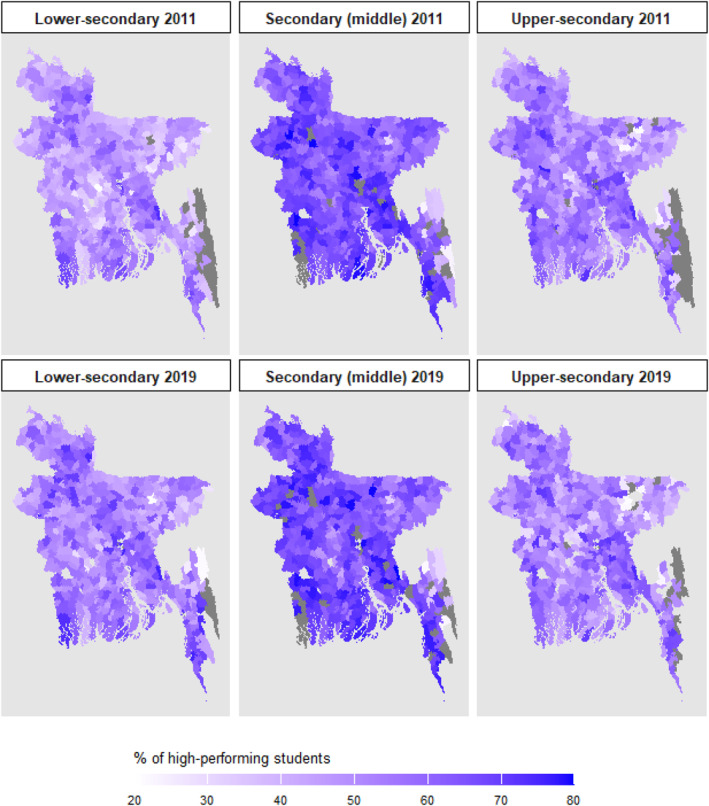
Average percentage of students scoring above 60% or grade ‘A−’ per school, aggregated by sub‐district (2011 and 2019). (1) The darker colour indicates a higher achievement level. (2) The outcome measure in the paper is the proportion of students achieving higher than 80% marks or A+ per school year. In these maps, I use a 60% cut‐off and aggregate the school mean at the sub‐district level to show a broader picture of achievement across countries. The marks, hence, include three different grade categories: ‘A+’ (80%–100%), ‘A’ (70%–79%), and ‘A−’ (60%–69%). *Source:* Author's calculations based on EMIS (n.d.).

As the maps in Figure 1 demonstrate, there are considerable geographical variations in students' performance in the national‐level exit exams at the secondary level, more so at the lower‐ and middle‐secondary levels. From two time points in 2011 and 2019, we can also see that the variance is quite consistent over time even almost one decade apart. The proportion of students achieving scores above 60% was 55% at the lower‐secondary level, 59% at the middle‐secondary level, and 50% at the upper‐secondary level in 2011, with similar figures observed in 2019. While it might be expected that the proportion of well‐achieving students would decrease as they progress to higher levels, the slightly higher figure at the middle‐secondary level before declining at the upper‐secondary level may partly be explained by the exclusion of vocational schools (a small proportion of all schools) from the analysis, as stated in the variable section, to maintain a focus on the general education track.

The research motivation is to primarily examine whether geographical variance in educational achievement in Bangladesh is due to differences in poverty by locality. The first research question investigates the association between local poverty and educational achievement. I then examine whether this association varies by schools' urbanicity. In the following parts, I describe the results according to these two research questions.

### 4.1 Local Poverty and Educational Achievement

**FIGURE 2:**
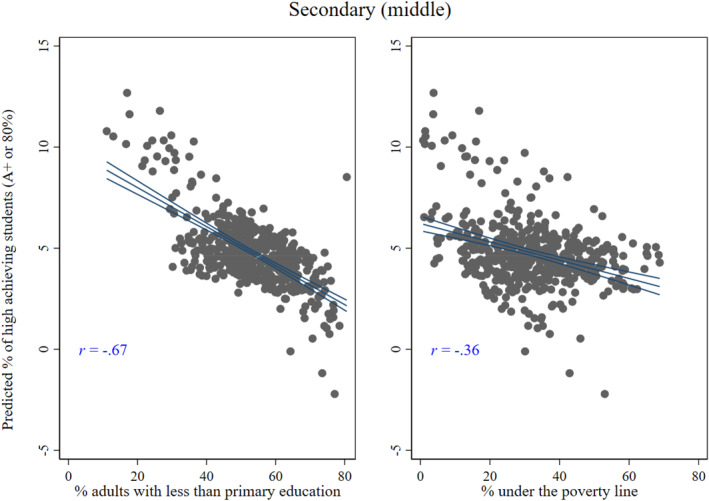
The inverse relationship of educational (left panel) and economic poverty (right panel) with higher achievement at the sub‐district level. (a) The predicted achievement ratio on the *y*‐axis is estimated based on model 7 in Table 2. I then take the subdistrict mean of achievement. The same plot for the lower‐ and upper‐secondary levels is presented in Supporting Information S1: Figures S3 and S4. (b) The *x*‐axis is limited up to 80% as a closer value is the higher bound or maximum value in the educational poverty variable in the left panel.

Results from Equation 1 suggest that educational poverty has a stronger and statistically significant negative association with educational achievement compared to economic poverty in subdistricts or localities. I find that for every percentage increase in educational poverty (or the proportion of adults with less than primary education in a local area), the proportion of students receiving A+ is likely to decrease by around 0.06% points (*p* < 0.001) at the middle‐secondary level, holding school characteristics and economic poverty constant. Results are similar for the lower‐ and upper‐secondary levels and thus are presented in Tables S2 and S3, respectively, of the supplementary materials. I plot the mean predicted achievement scores at the middle‐secondary level (from Model 7 in Table 2) by the subdistrict level against the mean of the two poverty measures in Figure 2. The graph illustrates the negative and stronger association for the educational poverty measure on the left panel. The same figure is also presented for the lower‐ and upper‐secondary levels in the supplementary materials (Supporting Information S1: Figures S3 and S4).

**TABLE 2:** The association between local poverty and educational achievement in secondary (middle) exit exam in Bangladesh, 2011–2019.

	Dependent variable: % Of students achieving A+ or 80% mark in a school year						
	(1)	(2)	(3)	(4)	(5)	(6)	(7)
	Baseline model	Economic poverty	Educational poverty	Both poverty	Economic poverty with urban variable	Educational poverty with urban variable	All variables
Level 3: Locality (sub‐district)							
Educational poverty (% adults below primary education)			−0.056***	−0.053***		−0.031**	−0.029**
			(0.011)	(0.012)		(0.010)	(0.011)
Economic poverty (% living under the poverty line)		−0.025*		−0.0058	−0.014		−0.0035
		(0.010)		(0.011)	(0.0098)		(0.010)
Level 2: School							
School has any computers? Yes (ref: no)		0.18*	0.18*	0.18*	0.18*	0.19*	0.19*
		(0.083)	(0.083)	(0.083)	(0.082)	(0.082)	(0.082)
School in an urban area (ref: rural)					2.32***	2.29***	2.29***
					(0.13)	(0.14)	(0.14)
Distance from sub‐district town (km)					−0.053***	−0.053***	−0.053***
					(0.0070)	(0.0070)	(0.0070)
School ownership (ref: autonomous/private)							
Government		−5.24**	−5.29**	−5.28**	−5.15**	−5.17**	−5.17**
		(1.93)	(1.92)	(1.92)	(1.90)	(1.90)	(1.90)
Local government		−9.08***	−9.11***	−9.11***	−8.92***	−8.94***	−8.94***
		(0.90)	(0.90)	(0.90)	(0.89)	(0.89)	(0.89)
Other		−2.12**	−2.10**	−2.10**	−1.87**	−1.85**	−1.86**
		(0.70)	(0.70)	(0.70)	(0.69)	(0.69)	(0.69)
Semi‐government		−8.79***	−8.73***	−8.73***	−8.12***	−8.08***	−8.08***
		(0.49)	(0.49)	(0.49)	(0.49)	(0.49)	(0.49)
School location (ref: flat land)							
Water‐inundated, riverbank and coastal areas		−0.96***	−0.88***	−0.88***	−0.63***	−0.59***	−0.59***
		(0.15)	(0.15)	(0.15)	(0.15)	(0.15)	(0.15)
Hilly areas (less developed communication infrastructures)		−0.78*	−0.73*	−0.74*	−0.43	−0.40	−0.41
		(0.36)	(0.36)	(0.36)	(0.36)	(0.36)	(0.36)
Year fixed effects	Yes	Yes	Yes	Yes	Yes	Yes	Yes
Basic controls	Yes	Yes	Yes	Yes	Yes	Yes	Yes
Constant	−1.94	9.20	12.5	12.5	7.34	9.10	9.10
	(12.1)	(12.0)	(12.1)	(12.1)	(12.0)	(12.0)	(12.0)
Random effects							
Level 4: District residual variance	2.07***	1.45	1.40	1.37	1.28	1.23	1.22
SE	(0.44)	(0.34)	(0.32)	(0.32)	(0.30)	(0.29)	(0.29)
*Variance explained (%)*		30	32	34	38	41	41
Level 3: Locality (sub‐district) residual variance	2.63***	2.36***	2.22***	2.23***	2.07***	2.03***	2.04***
SE	(0.23)	(0.21)	(0.21)	(0.21)	(0.19)	(0.19)	(0.19)
*Variance explained (%)*		10	16	15	22	23	23
							
Level 2: School residual variance	19.4***	18.7***	18.7***	18.7***	18.1***	18.1***	18.1***
SE	(0.29)	(0.29)	(0.29)	(0.29)	(0.28)	(0.28)	(0.28)
*Variance explained (%)*		4	4	4	7	7	7
Level 1: School‐year residual variance	119.7***	119.7***	119.7***	119.7***	119.7***	119.7***	119.7***
SE	(0.36)	(0.36)	(0.36)	(0.36)	(0.36)	(0.36)	(0.36)
*N* (Level 1—School‐year)	243,748						
*N* (Level 2—School)	24,275						
*N* (Level 3—Sub‐district)	505						
*N* (Level 4—District)	64						

*Note:* (a) The poverty measures are from 2011. (b) + sign in all models means variables added in addition to the previous model. (c) The baseline models (models 1 and 2) include year fixed effects and basic controls (the type of schools; whether the exam was for general or Islamic schools; whether schools only for boys, girls or for both; whether schools are registered as MPO; and the log of the total population in sub‐districts in the last census 2011). (d) MPO, a monthly pay order scheme to receive teachers' salaries from the government. (e) Variance explained compared to the baseline model or model 1. (f) *p < 0.05 **p < 0.01 ***p < 0.001.

The association between economic poverty and achievement is not significant at the middle‐secondary level. However, interestingly, economic poverty shows a significant association in Model 2 when it is entered alone in the model.^9^ But the significance disappears in Model 4 when it is added together with educational poverty. The inclusion of urbanicity in Model 6 slightly decreases the magnitude of the educational poverty coefficient but not the statistical significance. Besides, the inclusion of school resources such as having computers, distance from the town and schools' disadvantaged land location does not notably change the coefficients for the local poverty measures.

To assess the possibility of a non‐linear relationship between contextual poverty and achievement, I fit models using a quadratic function for both poverty measures. As shown in Supporting Information S1 Figure S5, a non‐linear association is evident for educational poverty, whereas the relationship for economic poverty appears linear. This finding indicates that educational poverty has a steeper negative association with achievement when the level of educational poverty is particularly high, but the relationship becomes less steep as the poverty level increases further. These results suggest that local areas with fewer role models or a significantly higher proportion of adults with low education may be particularly associated with poorer learning achievement in the next generation.

The results overall remain quite consistent both when using economic poverty measures from 2011 (as in Table 2 for the middle‐secondary level and Supporting Information S1: Tables S2 and S3 for the lower‐ and upper‐secondary levels) and from 2016 (Table S4 in the supplementary materials), as well as the measure in terms of ‘extreme poverty’ or the proportion of people below the lower national poverty line (Table S5 in the supplementary materials).

Considering the concern about the potential relationship between both poverty measures, I show in the correlation matrix in Table S1 of the supplementary materials that the two variables exhibit a mild association (*r* = 0.29). Similar to Figure 2, I also present two scatter plots in Supporting Information S1: Figures S3 and S4 showing the association between mean predicted achievement at the subdistrict level and both poverty measures at the lower‐ and upper‐secondary levels (using Model 7 in Supporting Information S1: Tables S2 and S3). The figures further illustrate a weaker association between economic poverty and achievement at the ecological level. I find a similar scenario when using predicted values from other models, that is, when educational poverty is not in the model (available upon request).

**FIGURE 3:**
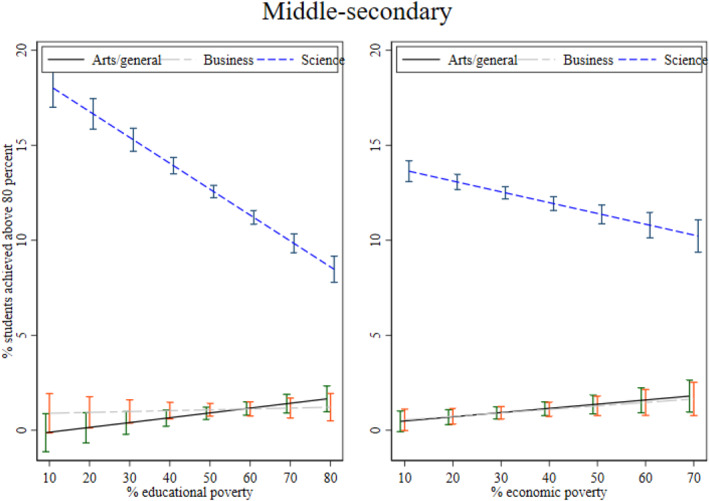
The inverse relationship between local poverty and achievement by academic stream.

**FIGURE 4:**
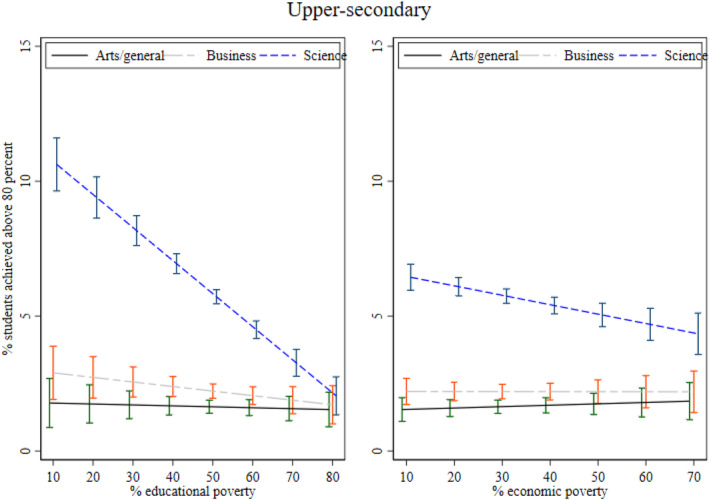
The inverse relationship between local poverty and achievement by academic stream.

***Heterogeneity by academic streams***. As I mentioned while introducing the first research question in the introduction, I also examine whether the association between local poverty and achievement varies by academic streams: arts/general, business, and science. I examine this mechanism at the middle‐ and upper‐secondary levels. I illustrate the interaction between academic streams and poverty in Figures 3 and 4. As expected, I find that the association between both poverty measures and achievement is stronger and significantly negative for the science stream compared to the arts/general and business streams. While significant, the slope is much steeper for science on educational poverty compared to economic poverty at both secondary levels. Thus, we see that economic poverty still matters for academic achievement in science but is lower than educational poverty. I posit that in local areas with greater educational poverty, pupils are more likely to struggle to find qualified teachers for supplementary tutoring, which is widely practised to improve achievement in science and mathematics in Bangladesh (Mahmud 2021). The role model effect is likely particularly significant for science subjects, as students' motivation to excel may be shaped by perceived returns to education, local employment opportunities, and the path dependency created by older generations who pursued science education and achieved success in professional life.

***Variance explained by the poverty measures***. I further examine to what extent local poverty explains achievement in schools. The random effects parts in Table 2 suggest that educational poverty explains more variance in educational achievement between subdistricts. Specifically, as Table 2 suggests, adding economic poverty to Model 2 along with other variables explains around 10% achievement variance at level 3 (subdistrict) compared to the baseline model. When I add educational poverty to Model 3, it explains a 21% variance. In the consecutive models, adding economic poverty back to the model does not explain any more variance than in Model 3. Similarly, adding the urban‐rural dummy to the models does not also explain further substantial variance compared to the variance explained by educational poverty. I observe similar scenarios in Supporting Information S1: Tables S2 and S3 for the lower‐ and upper‐secondary levels, respectively. I also find that educational poverty at the local/subdistrict level at level 3 explains more variance in achievement between districts at level 4 compared to the variance explained by economic poverty.

### 4.2 The Interaction Between School Urbanicity and Local Poverty

**FIGURE 5:**
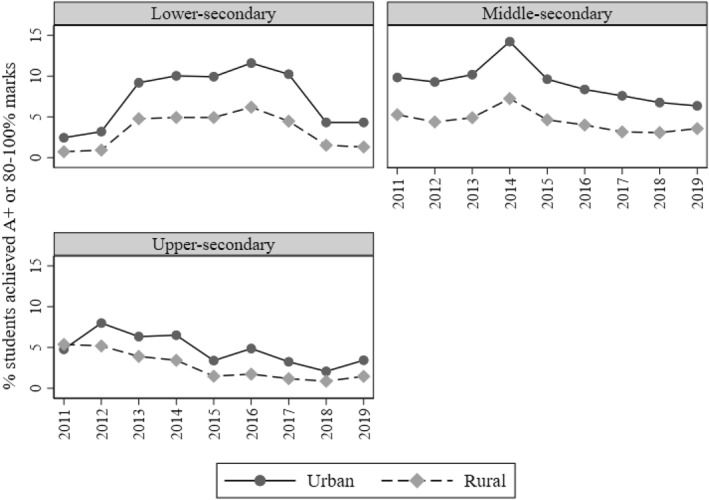
Urban‐rural differences in the proportion of high‐achieving students (achieving 80% or more marks) per school year, 2011–2019. Each secondary‐level category combines results from both general and Islamic (madrasah) schools. *Source:* Author's calculations based on EMIS (n.d.).

Figure 5 suggests that urban schools achieve significantly higher than rural schools at all secondary levels. This gap is wider at the lower‐ and middle‐secondary levels. I investigate whether the association between local poverty and achievement differs by school location in terms of urbanicity using Equation (2).

**FIGURE 6:**
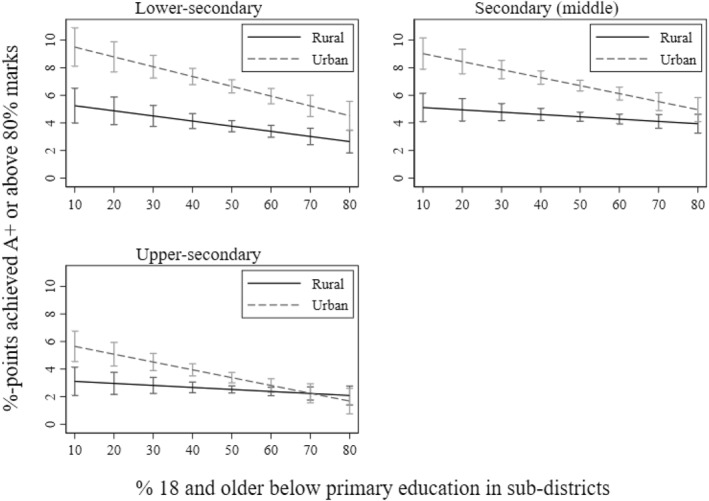
The interaction effect of educational poverty (adult education) and schools' urbanicity on educational achievement. The *x*‐axis is limited up to 80% as a closer value is the higher bound or maximum value in the variable.

I find that the association between educational poverty and achievement is downwardly steeper in urban schools compared to rural schools at all secondary levels. However, the urban school slope is flatter and non‐significant, especially for the middle‐ and upper‐secondary levels, on the association between economic poverty and achievement. Specifically, as Table 3 and Figure 6 illustrate, as the level of local poverty by education increases on the *x*‐axis at all three secondary levels, the slope for educational achievement in urban schools significantly decreases compared to rural schools. Only for the upper‐secondary level, does the slope become statistically non‐significant after local areas cross 60% of the educational poverty rate.

**TABLE 3:** The interplay between poverty and urban‐rural location and its association with students' achievement in secondary exit exams in, 2011–2019.

	Dependent variable: % Of students achieving A+ or 80% mark in a school year					
	(1)	(2)	(3)	(4)	(5)	(6)
	Lower‐secondary		Secondary (middle)		Upper‐secondary	
Educational poverty	−0.037**	−0.042**	−0.017	−0.026*	−0.015	−0.030**
	(0.014)	(0.014)	(0.012)	(0.011)	(0.012)	(0.011)
Economic poverty	0.0067	0.012	−0.0024	0.00024	−0.0020	−0.0018
	(0.013)	(0.013)	(0.010)	(0.010)	(0.0082)	(0.0088)
Urban	4.59***	4.23***	4.32***	3.08***	2.96***	1.16**
	(0.65)	(0.35)	(0.56)	(0.30)	(0.76)	(0.43)
**Urban × educational poverty**	−0.034**		−0.041***		−0.042**	
	(0.013)		(0.011)		(0.015)	
**Urban × economic poverty**		−0.042***		−0.025**		−0.0090
		(0.010)		(0.0088)		(0.012)
All controls	Yes	Yes	Yes	Yes	Yes	Yes
Year fixed effects	Yes	Yes	Yes	Yes	Yes	Yes
Constant	5.62	5.85	7.72	8.54	27.9***	29.2***
	(8.67)	(8.66)	(12.0)	(12.0)	(2.59)	(2.56)
*N* (level 1—School‐year)	94,856		243,748		44,948	
*N* (level 2—School)	23,856		24,275		5693	
*N* (level 3—Sub‐district)	505		505		499	
*N* (level 4—District)	64		64		64	

*Note:* (a) All controls include variables that are included in model 7 in Table 2, but not shown here in Table 3. (b) The models are estimated using Equation (2) with random effects, similar to analyses in the previous tables. The random effects part is excluded from Table 3 as the main parameters of interest here are the interaction terms. (c) The full models with other covariates are presented in Supporting Information S1: Table S6. *p < 0.05 **p < 0.01 ***p < 0.001.

The results align with my initial expectations that the achievement gap between urban schools in relatively advantaged and disadvantaged areas would be much stronger than that in rural areas. This finding reflects the structural and socioeconomic differences between urban and rural settings. Rural areas often consist of more homogenous socioeconomic groups due to a greater dependency on agriculture, resulting in less pronounced inequality within communities. In contrast, urban areas, including major cities, are characterised by stark inequalities, with a concentration of slums, informal settlements, and employment disparities. Schools in deprived urban areas tend to be of much lower quality, while wealthier neighbourhoods may benefit from access to well‐resourced schools. Moreover, as discussed in the theoretical section and elaborated in the discussion section below, urban areas with a significant slum population may face a shortage of role models, as the proportion of literate individuals is considerably lower compared to other parts of the country (BBS 2024).

**FIGURE 7:**
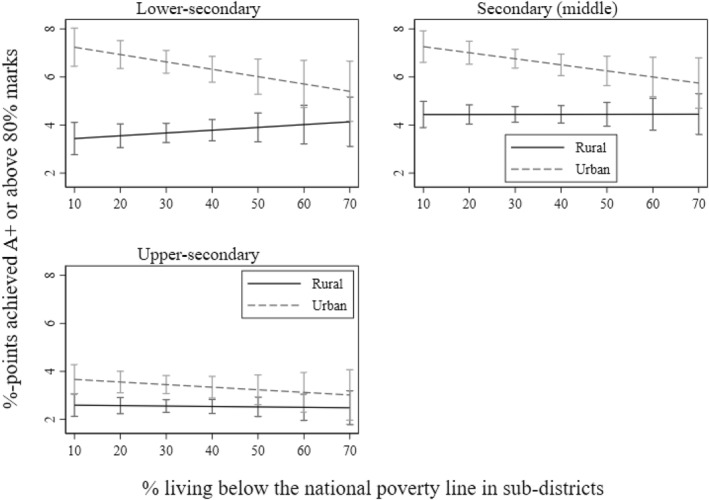
The interaction effect of economic poverty (living under the national poverty line) and schools' urbanicity on educational achievement. The *x*‐axis is limited up to 70% as a closer value is the higher bound or maximum value in the variable.

However, as illustrated in Table 3 and Figure 7, the negative association between economic poverty and achievement is significant in urban schools at the lower‐ and middle‐secondary levels. In both cases, the urban slope of economic poverty is flatter than that of the slope of educational poverty as the coefficient size in the interaction terms suggests. The urban slope does not significantly decrease at the upper‐secondary level as evident in its almost completely flat state.

### 4.3 Robustness

I conduct several robustness checks while highlighting the main ones. First, as aforementioned, since the main poverty measures are cross‐sectional and from 2011, I also use estimates for economic poverty from 2016 to see if the results are similar. As shown in Supporting Information S1: Table S4, the coefficients and significance level of the economic poverty variable remain quite comparable to that of estimates from 2011 in Table 2 and Supporting Information S1: Tables S2 and S3. However, due to the unavailability of data, I could not run the same regression for educational poverty (i.e., estimates from 2016).

*Second*, one may argue the 80% cut‐off point for academic achievement is arbitrary. However, when I run the same regression models using 70%–79% as the achievement cut‐off for the outcome variable, the findings remain consistent (see Supporting Information S1: Figure S6). Similarly, when I consider lower academic achievement (rather than high performance), I also get similar results. Precisely, when I use 50%–56% as the achievement in the outcome, I observe a similar pattern, but the coefficients are now positive (see Supporting Information S1: Figure S7). This means the more educational poverty a locality has the more prevalent lower‐level academic achievement is. The results remain non‐significant for economic poverty.

## 5 Discussion and Conclusion

In this paper, I show that poverty in local areas can have a strong negative association with learning achievement using the case of Bangladesh and employing original, untapped data. The literature on educational inequalities in LMICs has long debated whether family SES are more important than school characteristics for students' learning outcomes. Taking a step forward, this study suggests that in addition to school characteristics, the locality in which children grow up and socialise may also have effects on their achievement. There is a wealth of literature on this issue from advanced economies drawing this line of conclusion, but the evidence is quite limited from LMICs. I, however, was not able to compare the gradients of school and locality wealth covariates with that of family SES as the unit of measurement in the study is at the school level.

Overall, I find that educational poverty at the local or subdistrict level is significantly negatively associated with learning achievement at the secondary levels holding school characteristics and other relevant controls constant. In other words, having fewer adults with education in a locality is associated with lower academic performance. The association between economic poverty and achievement is mostly non‐significant. However, I find that both educational and economic poverty measures are associated with lower achievement for the science stream compared to the arts/general and business streams. The association is stronger for educational poverty.

The study provides important evidence regarding how poverty may matter for academic achievement in a low‐income setting compared to what the literature from high‐income countries shows. For instance, Ainsworth's (2002) important study from the US shows that neighbourhood poverty including its economic dimension may play a determining role in predicting educational outcomes. However, while this study focuses on aggregated neighbourhoods, I do not find economic poverty to be playing as influential role as educational poverty.

Aligning with the ‘role model effect’ of collective socialisation theory, as presented in the Theoretical Framework, I argue that less educational poverty (with a more educated community) in a local area creates a more congenial environment to encourage students to perform better. This suggests that learning takes place beyond the classroom in school (Bentley 2012). Educated older peers can act as good role models and reference groups, helping students set higher goals and achieve better outcomes (Kemper 1968). Conversely, communities with lower educational attainment may foster a discouraging environment, impeding students' aspirations.

Similar to other LMICs, Bangladesh has a relatively high young population compared to high‐income countries (World Bank, n.d.‐a) and has made notable progress in improving educational access, especially for marginalised groups (Asadullah et al. 2014). Thus, the educational attainment of the immediately preceding generation may play a path‐dependent role for the current generation within a community due to closer interpersonal communications (Crane 1991). Since the majority of secondary‐level schools in Bangladesh are financially supported by the government, individuals are less likely burdened by school fees. Hence, attending school may come down to being more aware of the importance of getting an education. Local role models may help with this process.

The stronger association between local poverty and learning achievement in the science stream further supports this argument that the presence of successful role models may positively influence science outcomes—an advantage often absent in poorer areas. As highlighted in the theoretical section, private tutoring is an established practice (Mahmud and Bray 2017), particularly for science students. Local areas with fewer educated adults as role models and a lack of qualified teachers are likely to have a detrimental influence on science stream performance.

Furthermore, the weaker association for economic poverty (including its overall null association) may be explained by the higher unemployment rates often faced by more higly‐educated young adults in Bangladesh (International Labour Organisation 2013). Hence, education does not consistently translate into greater economic resources due to a significant mismatch between skills and employment opportunities. Nevertheless, this educational background can still contribute to the generation of positive social capital, positively influencing younger, school‐age populations through beneficial socialisation processes (Bourdieu 1986; Coleman 1988). In addition, a sizeable proportion of the population, around 70%, are employed informally similar to many LMICs, without any job contract (International Labour Organisation 2018). Informally employed people tend to have a lower level of education while their earnings may be at the level of many contractual jobs with a higher level of education. Similarly, women's labour market integration is comparatively lower including educated women. This means education may distinguish the population much more than economic resources. The expansion of education in Bangladesh in terms of school enrolment has been a successful example thanks to its investment in the poorest, compared to countries with a similar development stage (Asadullah et al. 2014; Barkat‐e‐Khuda 2011). The unmatched educational poverty with economic poverty can also be observed in the moderate correlation between both variables in Supporting Information S1: Table S1. Despite this mismatch, individuals' education may have a positive influence on children's academic achievement in their communities, families and local areas through the collective socialisation process.

Moreover, I find that the association between local poverty and achievement differs by urban‐rural location, especially when I consider adult education as a measure of poverty. While urban areas generally achieve higher due to a greater concentration of educated individuals and role models in earlier generations, their achievement declines more sharply with increasing educational poverty. This greater sensitivity may be driven by structural inequalities, including concentrated poverty, the prevalence of slums, limited opportunities, and the absence of positive role models, which exacerbate disparities in educational opportunities (Coleman 1988; Harper et al. 2003). As noted in the theoretical section, poor urban areas, such as slums, often lack opportunities for children to interact with adults who can set examples to inspire educational and career aspirations. Conversely, rural areas demonstrate less sensitivity to educational poverty, potentially due to the ‘scarcity effect’ of role models. In rural contexts, the fewer role models available may have a disproportionately positive influence on students' aspirations, even in high‐poverty subdistricts.

The findings highlight the need for targeted interventions to address educational poverty in Bangladesh. Community‐based education programmes could leverage local social capital by engaging educated individuals as mentors or tutors, particularly in disadvantaged areas. Investments in science education, such as teacher training, laboratory facilities, and subsidised tutoring, could help mitigate resource disparities. Furthermore, addressing urban‐rural inequalities through improved teacher retention in rural areas and enhanced infrastructure in urban slums would ensure more equitable access to quality education. These interventions may provide a practical foundation for reducing educational inequalities in Bangladesh and similar LMIC contexts.

## 6 Limitations

I acknowledge several limitations in this study. First, the lack of micro‐level, individual data restricted the ability to explore how students' characteristics interact with those of schools and localities, including factors such as gender and ethnicity. For instance, early marriage may influence girls' education and their perceived returns to schooling, which could further vary by a locality's educational attainment among girls. However, this aspect could not be examined in detail due to the absence of individual‐level data.

While Bangladesh is largely an ethnically homogenous country, minority communities, particularly in the hilly regions, may experience unique educational challenges. Although I account for this issue in the analysis, it warrants further investigation in future research using individual‐level data. Despite these limitations, the study provides a comprehensive understanding of an issue that remains largely unexplored in LMICs, offering valuable insights for both academic and policy discussions.

Second, local spatial units could not be disaggregated any further than subdistricts, which would have helped get more insights into locality effects. Future research with more fine‐grained neighbourhood data from LMICs can examine to what extent the boundaries of smaller entities influence the educational outcomes of a community.

Third, the two estimates for local poverty are cross‐sectional while the outcome variable is time‐variant. The time‐varying poverty measures could have shown how changes in poverty are associated with changes in achievement. Despite these limitations, the wide coverage of geographical areas in Bangladesh provides a new understanding of what types of local poverty could be influential for educational outcomes in an LMIC setting. These findings could be relevant for many other LMICs with a notable presence of rural schools and very high urban inequality.

Fourth, this study's reliance on school‐level data presents a limitation in capturing the movement of high‐achieving and aspirational students between subdistricts in search of better schooling. This issue, relatively unexplored in literature on geographical variation in social and educational mobility (e.g., Breen and In 2024a, 2024b), is likely to be more relevant for better‐off households. While internal migration in Bangladesh tends to negatively impact education expenditure and school enrolment in poorer households (Ahmed et al. 2024), commuting or independent moves by students could particularly introduce bias into the results. As noted in the Theoretical Framework, students apply for upper‐secondary admissions based on their middle‐secondary results, and every subdistrict has access to upper‐secondary schools. However, without student‐level longitudinal data (currently unavailable to my knowledge), such movements remain untraceable. Future research could address this gap using data on internal migration histories.

Fifth, as noted in the Data and Methods section, this study captures over 90% of schools in Bangladesh, based on a rough estimation compared to statistics from the education ministry's website. While the proportion of missing schools is relatively low, it is important to acknowledge that the missing data may not be random. These gaps in the registered data could result from human error and may be associated with certain school characteristics.

## Supporting information

Supporting Information S1

## Data Availability

The data that support the findings of this study are available in the Education Management Information System, Bangladesh at http://emis.gov.bd/EMIS/IMS. These data were derived from the following resources available in the public domain: ‐ Education Management Information System, Bangladesh, http://emis.gov.bd/EMIS/IMS.

## Endnotes

^1^
Grades 6 to 8 are referred to as the ‘junior [lower] secondary’ level, while Grades 9 to 10 are known as the ‘secondary’ level or the first stage of upper secondary. Grades 11 to 12 constitute the upper‐secondary level. In this study, I use the term ‘middle‐secondary’ to specifically refer to the ‘secondary’ level, in order to provide a clear distinction. The government announced that there will no longer be any separate streams between arts, business, and science from 2022. However, this was reinstated in September 2024. See https://thefinancialexpress.com.bd/national/science‐arts‐and‐commerce‐streams‐to‐be‐reinstated‐in‐secondary‐education.

^2^
There are academic streams within schools at the middle‐ and upper‐secondary levels as I explain in the variable section. I control for these streams in the analysis and do not treat them as separate schools. Additionally, some schools have gender‐based sections, and academic performance data for boys and girls are reported separately, which I factor in while conducting the study.

^3^
Statistics from 2006 by the Intermediate and Secondary Education Boards, the Ministry of Education, Bangladesh (http://www.educationboard.gov.bd/edb_statistics.php), show that the total number of middle‐secondary schools was 23,789, which has likely increased since then.

^4^
Here, equivalent refers to the exam for students in Islamic schools (madrasah) at the same grade level called Junior Dakhil Certificate (JDC).

^5^
Here, equivalent refers to the exam for students in Islamic schools (madrasah) at the same grade level called Dakhil.

^6^
Here, equivalent refers to the exam for students in Islamic schools (madrasah) at the same grade level called Alim.

^7^
Most secondary schools in Bangladesh are private or semi‐government, which sometimes are categorised as private. However, I define them as semi‐government since these schools are mainly publicly funded while teachers are also recruited by the government. These schools can include local community members in school decision‐making processes. By contrast, private or autonomous schools in the category ‘a’ are fully private.

^8^
Each subdistrict comprises a mix of all these schools. Thus, students graduating from category ‘a’ schools that offer education only up to the middle‐secondary level may transition to other schools within the area that provide upper‐secondary education. Their likelihood of moving to a different subdistrict may depend on factors such as their motivation and socioeconomic status, which cannot be captured in the school‐level data used in this study. The potential migration mechanisms are further elaborated in the limitations section.

^9^
A similar trend is observed at the upper‐secondary level (Table S3 in the supplementary materials). However, economic poverty is not significantly associated with achievement at the lower‐secondary level in any of the models as shown in Supporting Information S1: Table S2.
