# Supplementary material for: The Link Between Contextual Poverty and Academic Achievement: Evidence Using Panel Data From a Lower‐Middle‐Income Country
Source: Br J Sociol. 2025 Mar 26;76(4):725–43. doi: 10.1111/1468-4446.13208 (PMC12412087; doi:10.1111/1468-4446.13208)
Supplement: Supplementary file 1 — Supporting Information S1 [file BJOS-76-725-s001.docx]

**Supporting Information**

**Figure S1**. The relationship between educational poverty (% adults below primary education) and economic poverty in 2011 and 2016 at the sub-district level.


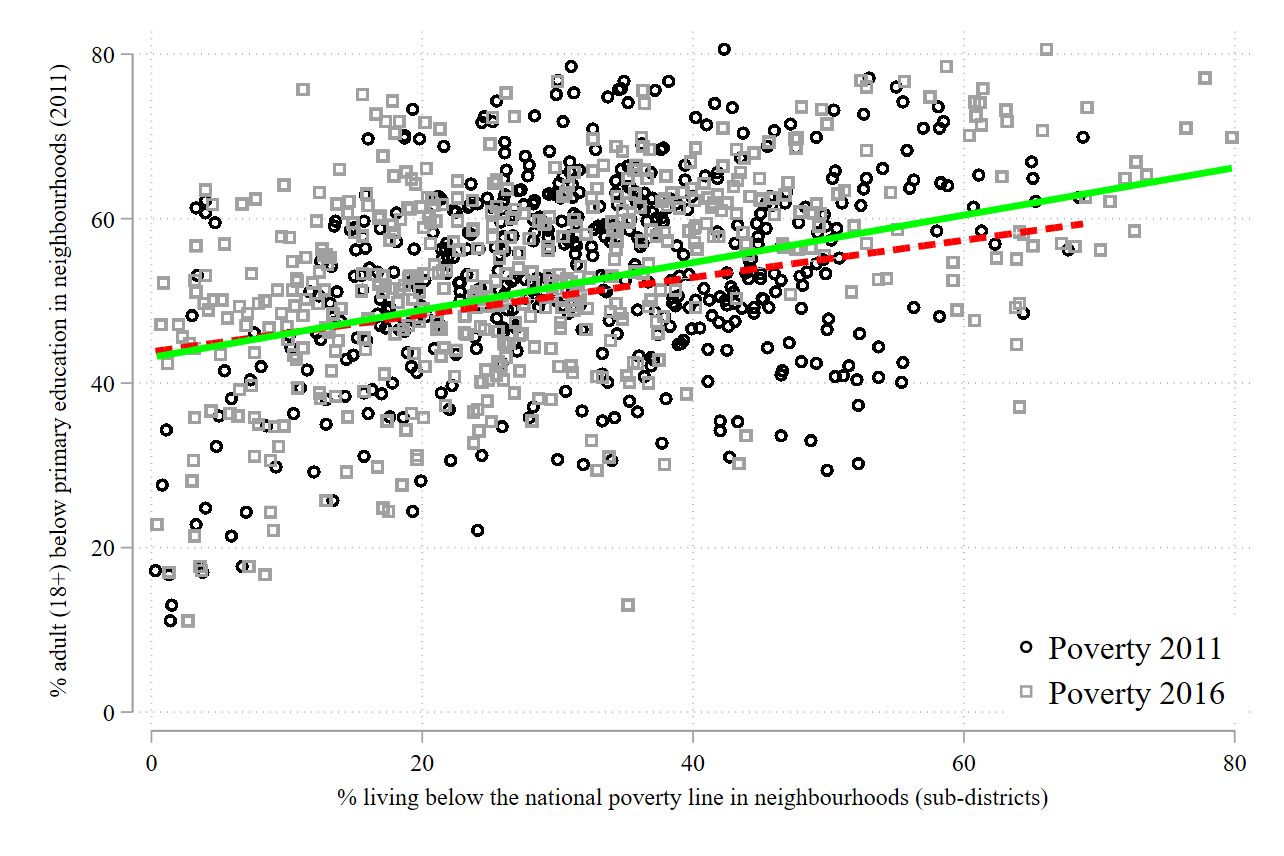


*Notes*: (a) The dashed fitted line represents the relationship for the economic poverty measure from 2011 while the solid fitted line signifies it for the economic poverty measure from 2016. (b) The x-axis indicates economic poverty (% of people living below the national poverty line).

Source: EMIS (n.d.) and (World Bank, 2016).

**Figure S2**. The relationship between the economic poverty estimates in 2011 and 2016 at the sub-district level.


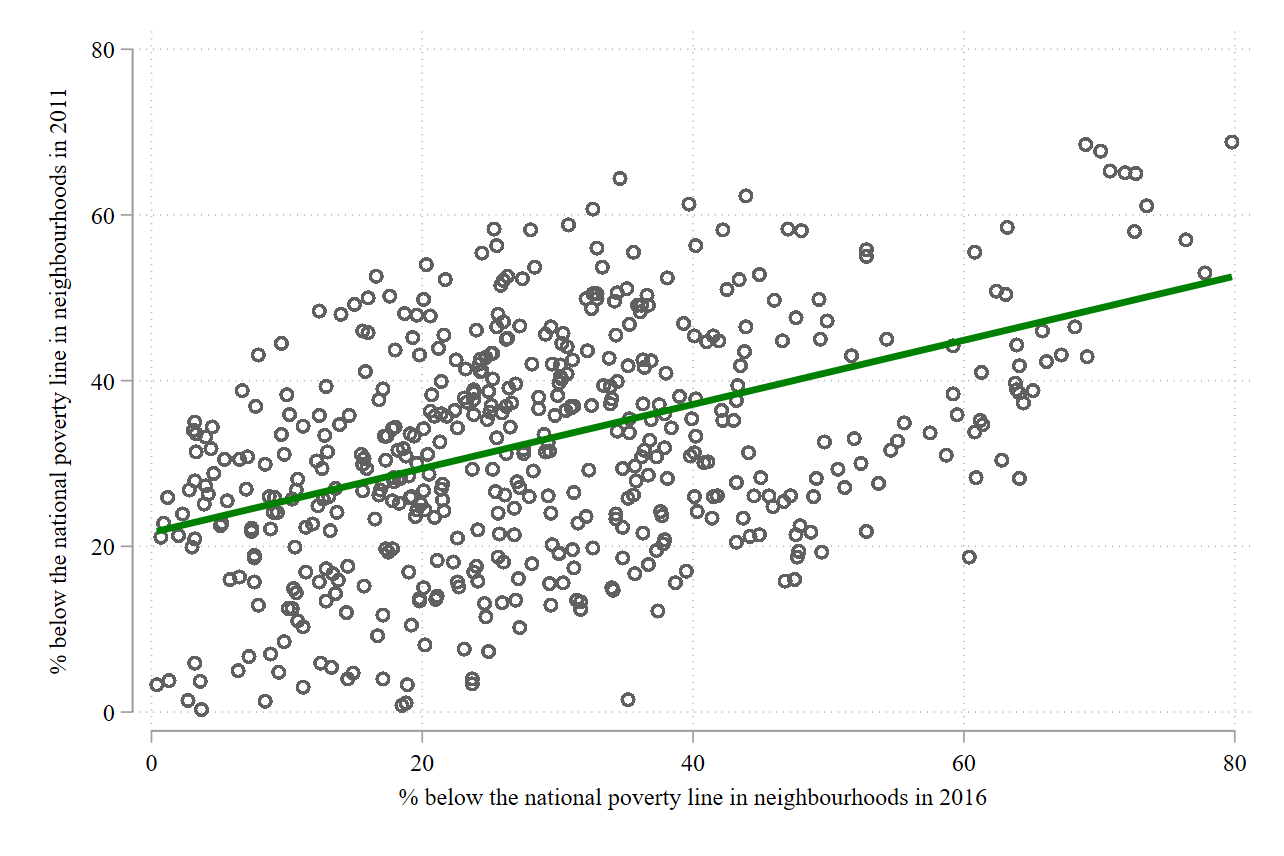


*Notes*: Both the x- and y-axis indicate economic poverty (% of people living below the national poverty line).

Source: EMIS (n.d.) and (World Bank, 2016).

**Table S1**. Correlation matrix of locality and school poverty indicators

|  | Educational poverty (% with less than primary education) (2011) | Economic poverty (% living under the poverty line) (2011) | Economic poverty (% living under the poverty line) (2016) | Ratio of the households without toilets | Schools with computer | Urban school | School distance from town |
| --- | --- | --- | --- | --- | --- | --- | --- |
| Educational poverty (% with less than primary education) (2011) | 1 |  |  |  |  |  |  |
| Economic poverty (% living under the poverty line) (2011) | 0.29 | 1 |  |  |  |  |  |
| Economic poverty (% living under the poverty line) (2016) | 0.40 | 0.49 | 1 |  |  |  |  |
| Ratio of the households without toilets | 0.42 | 0.07 | 0.47 | 1 |  |  |  |
| Schools with computer | -0.0023 | -0.0015 | -0.0085 | -0.013 | 1 |  |  |
| Urban school | -0.29 | -0.15 | -0.12 | -0.18 | 0.0068 | 1 |  |
| School distance from town | 0.13 | 0.066 | 0.066 | 0.10 | 0.0009 | -0.45 | 1 |

**Figure S3.** The inverse relationship of educational (left panel) and economic poverty (right panel) with higher achievement at the sub-district level


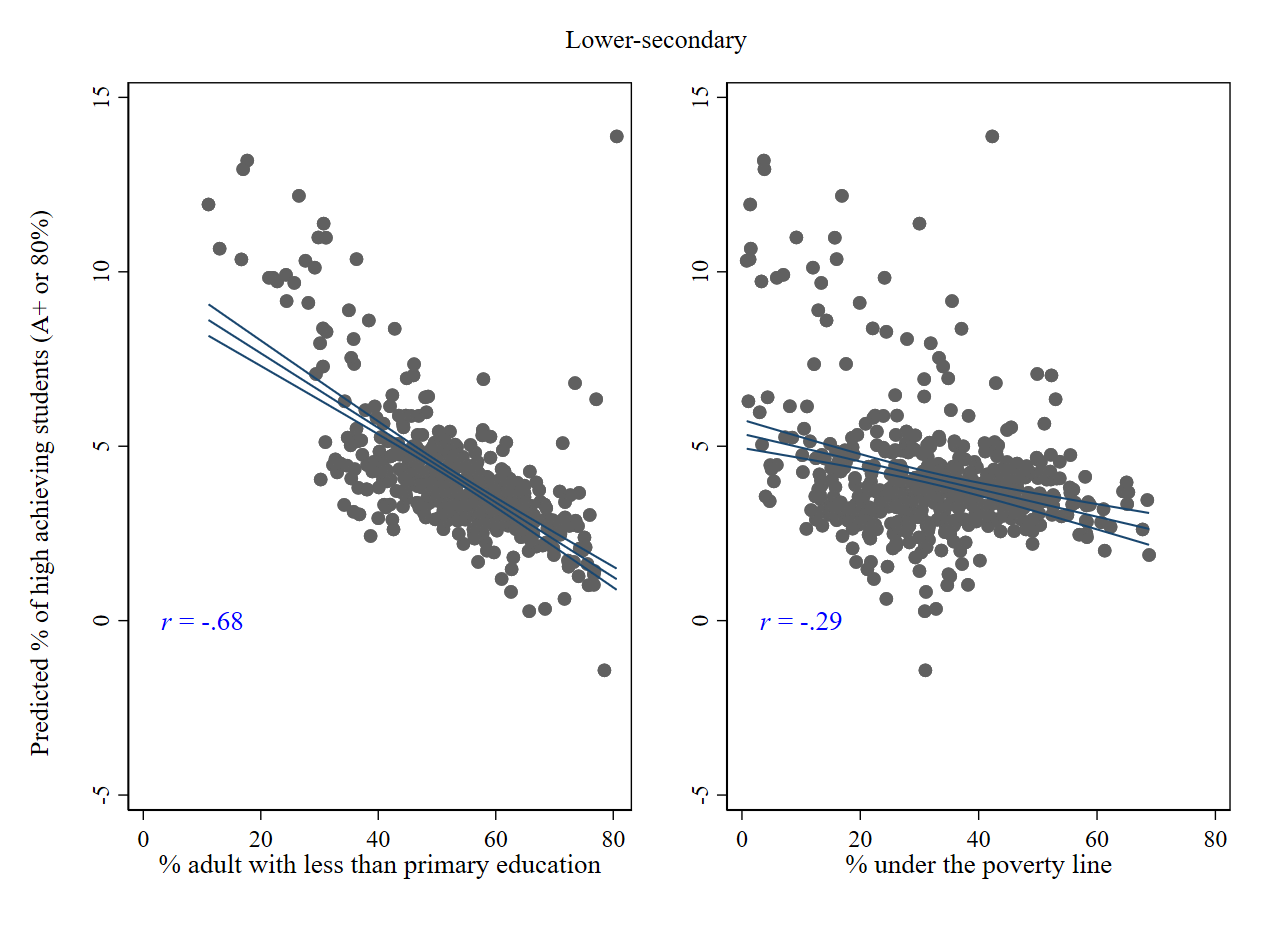


**Figure S4.** The inverse relationship of educational (left panel) and economic poverty (right panel) with higher achievement at the sub-district level

**
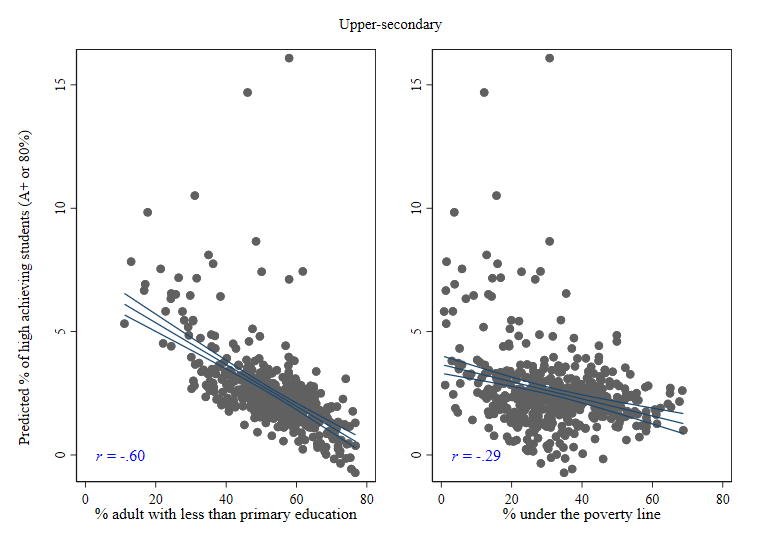
**

**Figure S5.** The association between local poverty and achievement considering squared terms of both poverty measures.


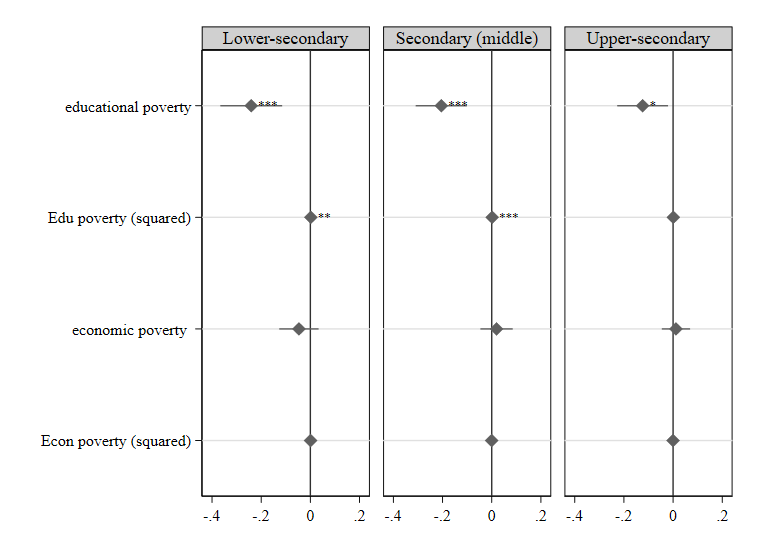


* p<0.05 ** p<0.01 *** p<0.001.

**Table S2**. The association between local poverty and educational achievement in **lower-secondary** exit exam in Bangladesh, 2011-2019.

|  | Dependent variable: % of students in a school received A+ or 80% mark in exit exam | | | | | | | |
| --- | --- | --- | --- | --- | --- | --- | --- | --- |
|  | (1) | (2) | (3) | (4) | (5) | (6) | (7) | |
|  | Baseline model | economic poverty | education poverty | both poverty | economic poverty with urban | education poverty with urban | All variables | |
| **Level 3: Locality (sub-district)** |  |  |  |  |  |  |  | |
| Educational poverty (% adult below primary education) |  |  | -0.074*** | -0.076*** |  | -0.045*** | -0.047*** | |
|  |  |  | (0.013) | (0.014) |  | (0.013) | (0.014) | |
| Economic poverty (% living under the poverty line) |  | -0.025 |  | 0.0036 | -0.012 |  | 0.0058 | |
|  |  | (0.013) |  | (0.013) | (0.012) |  | (0.013) | |
| Year fixed effects | Yes | Yes | Yes | Yes | Yes | Yes | Yes | |
| Basic controls | Yes | Yes | Yes | Yes | Yes | Yes | Yes | |
| Constant | -9.83 | 5.41 | 10.0 | 10.0 | 3.89 | 6.74 | 6.73 | |
|  | (8.81) | (8.71) | (8.73) | (8.73) | (8.63) | (8.67) | (8.67) | |
| **Random effects** |  |  |  |  |  |  |  | |
| Level 4: District residual variance | 2.29*** | 2.37*** | 1.92** | 1.92** | 2.15*** | 1.86** | 1.85* | |
|  | (0.52) | (0.54) | (0.46) | (0.46) | (0.50) | (0.45) | (0.44) | |
| *Variance explained (%)* |  | -3 | 16 | 16 | 6 | 19 | 19 | |
| Level 3: Locality (sub-district) residual variance | 3.95*** | 3.45*** | 3.29*** | 3.29*** | 3.16*** | 3.14*** | 3.14*** | |
|  | (0.34) | (0.31) | (0.29) | (0.29) | (0.28) | (0.28) | (0.28) | |
| *Variance explained (%)* |  | 13 | 17 | 17 | 20 | 20 | 20 | |
| Level 2: School residual variance | 31.5*** | 29.9*** | 29.9*** | 29.9*** | 29.2*** | 29.2*** | 29.2*** | |
|  | (0.40) | (0.38) | (0.38) | (0.38) | (0.38) | (0.38) | (0.38) | |
| *Variance explained (%)* |  | 5 | 5 | 5 | 7 | 7 | 7 | |
| Level 1: School-year residual variance | 36.9*** | 36.9*** | 36.9*** | 36.9*** | 36.9*** | 36.9*** | 36.9*** | |
|  | (0.20) | (0.20) | (0.20) | (0.20) | (0.20) | (0.20) | (0.20) | |
| *N* (Level 1 – School-year) | 94,856 | | | | | | |  |
| *N* (Level 2 – School) | 23,856 | | | | | | |  |
| *N* (Level 3 – Sub-district) | 505 | | | | | | |  |
| *N* (Level 4 – District) | 64 | | | | | | |  |

*Notes*: (a) The poverty measures are from 2011. (b) + sign in all models means variables added in addition to the previous model. (c) The baseline models (models 1 and 2) include year fixed effects and basic controls (the type of schools; whether the exam was for general or Islamic schools; whether schools only for boys, girls or for both; whether schools are registered as MPO; and the log of the total population in sub-districts in the last census 2011). (d) MPO, a monthly pay order scheme to receive teachers’ salaries from the government. (e) Variance explained compared to the baseline model or model 1, respectively. (f) All other control variables as in Table 2 of the paper have been omitted from this table but can be made available upon request. (g) * p<0.05 ** p<0.01 *** p<0.001.

**Table S3**. The association between local poverty and educational achievement in **upper-secondary** exit exam in Bangladesh, 2011-2019.

|  | Dependent variable: % of students achieved A+ or 80% mark in a school year | | | | | |  |
| --- | --- | --- | --- | --- | --- | --- | --- |
|  | (1) | (2) | (3) | (4) | (5) | (6) | (7) |
|  | Baseline model | economic poverty | education poverty | both poverty | economic poverty with urban | education poverty with urban | All variables |
| **Level 3: Locality (sub-district)** |  |  |  |  |  |  |  |
| Educational poverty (% adult below primary education) |  |  | -0.041*** | -0.038*** |  | -0.032** | -0.031** |
|  |  |  | (0.0099) | (0.011) |  | (0.0099) | (0.010) |
| Economic poverty (% living under the poverty line) |  | -0.017* |  | -0.0068 | -0.012 |  | -0.0040 |
|  |  | (0.0084) |  | (0.0085) | (0.0083) |  | (0.0084) |
| School registered as MPO | Yes | Yes | Yes | Yes | Yes | Yes | Yes |
| Year fixed effects | Yes | Yes | Yes | Yes | Yes | Yes | Yes |
| Basic controls | Yes | Yes | Yes | Yes | Yes | Yes | Yes |
| Constant | 11.0*** | 28.2*** | 30.2*** | 30.2*** | 27.9*** | 29.4*** | 29.4*** |
|  | (2.52) | (2.53) | (2.56) | (2.56) | (2.51) | (2.54) | (2.54) |
| **Random effects** |  |  |  |  |  |  |  |
| Level 4: District residual variance | 0.84 | 0.52* | 0.41* | 0.40* | 0.48* | 0.38** | 0.38* |
| SE | (0.22) | (0.17) | (0.15) | (0.15) | (0.16) | (0.14) | (0.14) |
| *Variance explained (%)* |  | 38 | 52 | 52 | 43 | 54 | 54 |
|  |  |  |  |  |  |  |  |
| Level 3: Locality (sub-district) residual variance | 1.00 | 0.85 | 0.82 | 0.82 | 0.77 | 0.77 | 0.76 |
| SE | (0.22) | (0.20) | (0.20) | (0.20) | (0.19) | (0.19) | (0.19) |
| *Variance explained (%)* |  | 15 | 18 | 19 | 23 | 23 | 24 |
|  |  |  |  |  |  |  |  |
| Level 2: School residual variance | 19.6*** | 17.7*** | 17.7*** | 17.7*** | 17.6*** | 17.6*** | 17.6*** |
| SE | (0.52) | (0.48) | (0.48) | (0.48) | (0.48) | (0.48) | (0.48) |
| *Variance explained (%)* |  | 10 | 10 | 10 | 10 | 10 | 10 |
| Level 1: School-year residual variance | 47.6*** | 47.6*** | 47.6*** | 47.6*** | 47.6*** | 47.6*** | 47.6*** |
| SE | (0.34) | (0.34) | (0.34) | (0.34) | (0.34) | (0.34) | (0.34) |
| *N* (Level 1 – School-year) | 44,948 | | | | | |  |
| *N* (Level 2 – School) | 5,693 | | | | | |  |
| *N* (Level 3 – Sub-district) | 499 | | | | | |  |
| *N* (Level 4 – District) | 64 | | | | | |  |

*Notes*: The notes in Table S2 apply here in Table S3 as well. SE, standard error. * p<0.05 ** p<0.01 *** p<0.001.

**Table S4**. The association between local poverty and educational achievement at the secondary levels while considering the economic poverty measure from 2016.

|  | Dependent variable: % of students in a school received A+ (80-100% mark) in exit exams | | | | | | | | |
| --- | --- | --- | --- | --- | --- | --- | --- | --- | --- |
|  | Lower-secondary | | | Secondary (middle) | | | Upper-secondary | | |
|  | (LS1) | (LS2) | (LS3) | (S1) | (S2) | (S3) | (US1) | (US2) | (US3) |
|  | Baseline model | +Local poverty | + School background | Baseline model | + Local poverty | + School background | Baseline model | + Local poverty | + School background |
| **Level 3: Locality (sub-district)** |  |  |  |  |  |  |  |  |  |
| Educational poverty (2011) | | -0.099*** | -0.045*** |  | -0.072*** | -0.026*** |  | -0.047*** | -0.029** |
|  |  | (0.014) | (0.013) |  | (0.011) | (0.011) |  | (0.011) | (0.010) |
| Economic poverty (2016) | | -0.009 | -0.0005 |  | -0.0059 | -0.014 |  | -0.0096 | -0.013 |
|  |  | (0.012) | (0.012) |  | (0.01) | (0.0094) |  | (0.0086) | (0.0080) |
| Constant | -9.83 | -2.58 | 6.72 | -1.94 | 4.26 | 9.55 | 11.0*** | 15.1*** | 34.1*** |
|  | (8.81) | (8.83) | (8.68) | (12.1) | (12.1) | (12.0) | (2.52) | (2.53) | (1.32) |
| *N* (Level 1 – School-year) | 94,856 | | | 243,748 | | | 44,948 | | |
| *N* (Level 2 – School) | 23,856 | | | 24,275 | | | 5,693 | | |
| *N* (Level 3 – Sub-district) | 505 | | | 505 | | | 499 | | |
| *N* (Level 4 – District) | 64 | | | 64 | | | 64 | | |

*Notes*: (a) + sign in all models means variables added in addition to the previous model. (b) Models described as: LS, lower secondary; S (middle), secondary; and US, upper-secondary. (c) The baseline models (models 1 and 2) include year fixed effects and basic controls (the type of schools; whether the exam was for general or Islamic schools; academic streams—humanities, business, and science— apart from secondary (middle); and the log of the total population in sub-districts in the last census 2011). (d) MPO, a monthly pay order scheme to receive teachers’ salaries from the government. (e) Variance explained compared to the baseline model or models 1a and 2a for lower secondary and secondary (middle), respectively. (f) All other control variables as in Table 2 of the paper have been omitted from this table but can be made available upon request. (g) * p<0.05 ** p<0.01 *** p<0.001.

**Table S5**. The association between local poverty and educational achievement while considering economic poverty as ‘extreme poverty’.

|  | Dependent variable: % of achieving A+ or 80% mark in a school year | | |
| --- | --- | --- | --- |
|  | (1) | (2) | (3) |
|  | Lower-secondary | Secondary (middle) | Upper-secondary |
| **Level 3: Locality (sub-district)** |  |  |  |
| Educational poverty (% adult below primary education) | -0.49*** | -0.29*** | -0.030** |
|  | (0.013) | (0.011) | (0.010) |
| Economic poverty (% living under the lower poverty line) | 0.014 | -0.0077 | -0.0088 |
|  | (0.016) | (0.013) | (0.011) |
| Year fixed effects |  |  |  |
| Basic controls | Yes | Yes | Yes |
| Constant | 6.75 | 9.08 | 29.5*** |
|  | (8.67) | (12.0) | (2.54) |
| School-year N | 94856 | 243748 | 44948 |

*Notes*: (a) All other control variables as in Table 2 of the paper and the random effects part have been omitted from this table but can be made available upon request. (b) * p<0.05 ** p<0.01 *** p<0.001.

**Table S6**. The interaction between poverty and urban-rural location and its association with students’ achievement in secondary exit exams (full results of Table 2).

|  | Dependent variable: % of students achieved A+ or 80% mark in a school year | | | | | |
| --- | --- | --- | --- | --- | --- | --- |
|  | Lower-secondary | | Secondary (middle) | | Upper-secondary | |
|  | (1) | (2) | (3) | (4) | (5) | (6) |
|  |  |  |  |  |  |  |
| Educational poverty | -0.037** | -0.042** | -0.017 | -0.026* | -0.015 | -0.030** |
|  | (0.014) | (0.014) | (0.012) | (0.011) | (0.012) | (0.011) |
| Economic poverty | 0.0067 | 0.012 | -0.0024 | 0.00024 | -0.0020 | -0.0018 |
|  | (0.013) | (0.013) | (0.010) | (0.010) | (0.0082) | (0.0088) |
| Urban (ref: rural school) | 4.59*** | 4.23*** | 4.32*** | 3.08*** | 2.96*** | 1.16** |
|  | (0.65) | (0.35) | (0.56) | (0.30) | (0.76) | (0.43) |
| Urban × Educational poverty | -0.034** |  | -0.041*** |  | -0.042** |  |
|  | (0.013) |  | (0.011) |  | (0.015) |  |
| Urban × Economic poverty |  | -0.042*** |  | -0.025** |  | -0.0090 |
|  |  | (0.010) |  | (0.0088) |  | (0.012) |
| Constant | 5.62 | 5.85 | 7.72 | 8.54 | 27.9*** | 29.2*** |
|  | (8.67) | (8.66) | (12.0) | (12.0) | (2.59) | (2.56) |
| **Random effects** |  |  |  |  |  |  |
| Level 4: District residual variance | 1.84* | 1.85** | 1.18 | 1.20 | 0.35** | 0.38** |
|  | (0.44) | (0.44) | (0.28) | (0.29) | (0.14) | (0.14) |
| Level 3: Locality (sub-district) residual variance | 3.09*** | 3.06*** | 1.99*** | 2.00*** | 0.77 | 0.76 |
|  | (0.28) | (0.28) | (0.19) | (0.19) | (0.19) | (0.19) |
| Level 2: School residual variance | 29.2*** | 29.2*** | 18.1*** | 18.1*** | 17.6*** | 17.6*** |
|  | (0.38) | (0.38) | (0.28) | (0.28) | (0.48) | (0.48) |
| Level 1: School-year residual variance | 36.9*** | 36.9*** | 119.7*** | 119.7*** | 47.6*** | 47.6*** |
|  | (0.20) | (0.20) | (0.36) | (0.36) | (0.34) | (0.34) |
| School year | 94856 | 94856 | 243748 | 243748 | 44948 | 44948 |

*Notes*: All other control variables as in Table 2 of the paper have been omitted from this table but can be made available upon request. * p<0.05 ** p<0.01 *** p<0.001.

**Figure S6.** The association between local poverty and educational achievement (% students achieved 70-79 % marks) at three different exit exams at the secondary level in Bangladesh, 2011-2019.

**
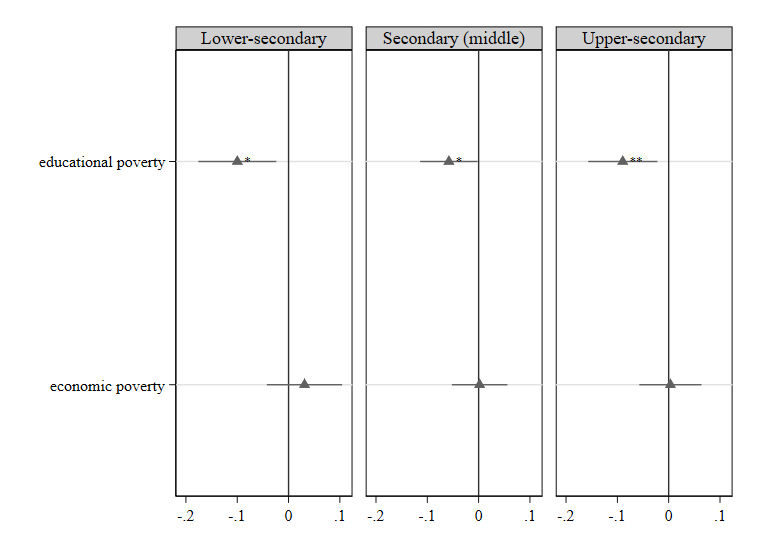
**

***Notes***. The results are derived from the same equation as equation 1, but the cut-off value for the outcome variable or academic achievement is from 70 to 79 percent. * p<0.05 ** p<0.01 *** p<0.001.

**Figure S7.** The association between local poverty and educational achievement (% students achieved 50-59 % or a B grade) at three different exit exams at the secondary level in Bangladesh, 2011-2019.

**
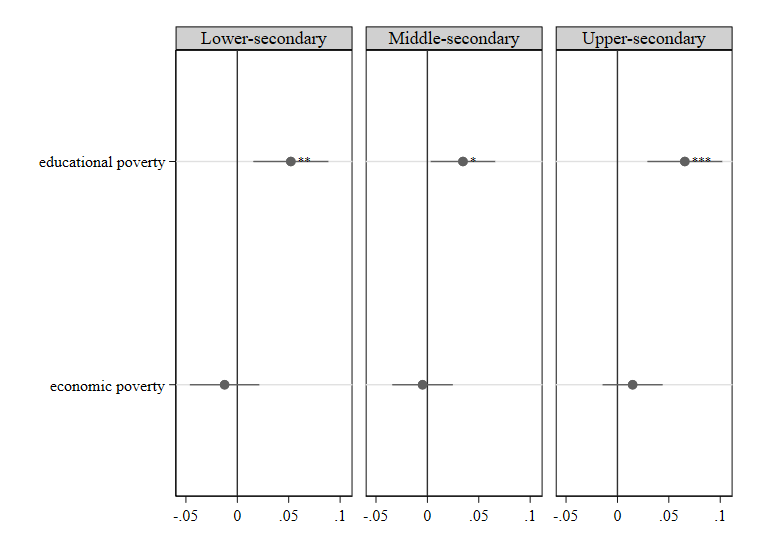
**

***Notes***. The results are derived from the same equation as equation 1, but the cut-off value for the outcome variable or academic achievement is between 50 and 59 percent. * p<0.05 ** p<0.01 *** p<0.001.
